# Toxicity and Feeding Deterrent Effect of 2-Methylanthraquinone from the Wood Extractives of *Tectona grandis* on the Subterranean Termites *Coptotermes formosanus* and *Reticulitermes speratus*

**DOI:** 10.3390/insects7040063

**Published:** 2016-11-08

**Authors:** Maya Ismayati, Akiko Nakagawa-Izumi, Nadia Nuraniya Kamaluddin, Hiroshi Ohi

**Affiliations:** 1 Graduate School of Life and Environmental Sciences, University of Tsukuba, Tsukuba 305-0872, Japan; maya_ismayati@biomaterial.lipi.go.id (M.I.); nadia.kamaluddin@gmail.com (N.N.K.); oi.hiroshi.gm@tsukuba.ac.jp (H.O.); 2 Research Center for Biomaterial, Indonesian Institute of Sciences (LIPI), Jl. Raya Bogor km. 46 Cibinong, Bogor 16911, Indonesia

**Keywords:** *Tectona grandis*, 2-methylanthraquinone, *Coptotermes formosanus*, *Reticulitermes speratus*

## Abstract

No-choice feeding tests using ethanol, chloroform, and acetone extractives of teak (*Tectona grandis*) heartwood clearly showed feeding deterrent activity and toxicity to the subterranean termite *Reticulitermes speratus*. The amount of 2-methylanthraquinone (MAQ) in teak wood extractives was not related to the feeding deterrents or toxicity, as shown by the no-choice feeding tests conducted using crude extractives containing various amounts of MAQ, MAQ alone, and fractions of crude extractives. As a native pest, the subterranean termite *Coptotermes formosanus* was more tolerant to the fractions of crude extractives than *Reticulitermes speratus*, and the mortality observed in *C. formosanus* was not due to the presence of MAQ.

## 1. Introduction

Preservatives extracted from natural products are important with respect to the effects of their extractives on wood durability. Teak (*Tectona grandis*) is a valuable wood with high durability. The naturally occurring anthraquinone, 2-methylanthraquinone (MAQ) found in teak heartwood is often associated with its high durability [1,2]. According to several studies, teak wood extractives are fundamental in its resistance against termites [1,2,3] and fungi [1,2,4]. There is a good correlation between termite resistance and tree age and extractive contents of teak wood [1]. Teak wood extractives and several fractions obtained from ethyl acetate extraction have strongly deterred *Reticulitermes speratus* [3]. MAQ is possibly an important compound providing durability to teak wood against termite infestations [5] and fungi [6]. At high concentrations, MAQ is toxic to the termite *R. flavipes* and acts as a repellent to the drywood termite *Cryptotermes brevis* [7]. In contrast, it is non-toxic to subterranean termites *Coptotermes lacteus* and *Nasutitermes exitiosus*, merely acting as a deterrent [1,2]. Teak sapwood is generally considered less durable; a recent finding of MAQ in sapwood, which may not be present at effective levels, suggested that this compound would not affect the wood durability [8].

However, most of these studies have focused on the effects of such compounds within trees, and there is limited information on termite resistance in relation to the amount of MAQ in teak wood extractives. We hypothesized that wood resistance against termites depends on wood compounds and contents, i.e., certain compounds provide resistance to termites, even at low concentrations. Thus, the MAQ content of teak wood and its extractives was determined in this study; we also evaluated the activity of MAQ and teak wood extractives against two species of subterranean termites, *Coptotermes formosanus* and *Reticulitermes speratus*.

## 2. Materials and Methods

### 2.1. Termites and T. grandis Heartwood Sampling

*R. speratus* was collected from the Living Sphere Simulation Field, Research Institute for Sustainable Humanosphere (RISH), Kyoto University (Kagoshima Prefecture, Japan) and maintained at the University of Tsukuba (Ibaraki Prefecture, Japan), under controlled laboratory conditions (28 °C and 80% relative humidity). *C. formosanus* was maintained at the Forestry and Forest Products Research Institute (FFPRI), Ibaraki, Japan.

Debarked disks of *T. grandis* natural wood imported from Myanmar in 2000 was kindly provided by the HOKUSAN Corporation Ibaraki Factory (Ibaraki Prefecture, Japan). A radius of the wood disk was 35 cm, and lengths of sapwood and heartwood parts were 2.5 cm and 32.5 cm, respectively. The number of the annual rings was counted to 210.

### 2.2. Extraction and Fractionation

**Figure 1:**
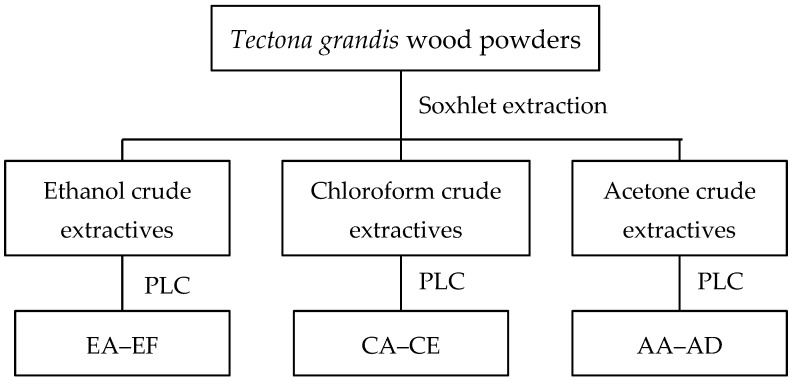
Schematic representation of the extraction and fractionation performed for *T. grandis* wood extractives. Notes: PLC: Preparative layer chromatography (Silica gel 60 F_254_ 2 mm, 20 cm × 20 cm); EA–EF = fractions obtained from ethanol crude extractives; CA–CE = fractions obtained from chloroform crude extractives; AA–AD = fractions obtained from acetone crude extractives.

Wood powder (2 g, oven-dried weight) was sieved (40–60 mesh size, 180–355 μm opening) and Soxhlet-extracted for 8 h using 150 mL of ethanol, chloroform, or acetone, respectively. All solvents were a special grade from Wako Pure Chemical Industries, Japan. Three replicates were performed for each extraction and the resulting solvents were evaporated to a concentrate and crude extractives were obtained. Approximately 100 mg of each crude extractive was dissolved in 0.5 mL of each solvent and fractionated by preparative layer chromatography (PLC) (PLC Silica gel 60 F_254_ 2 mm, 20 cm × 20 cm, Merck KGaA, Darmstadt, Germany). The mobile phase used for ethanol, chloroform, and acetone crude extractives was *n*-hexane: ethyl acetate (9 mL: 1 mL). Extraction and fractionation are schematically represented in Figure 1. Each fraction (EB–EF, CA–CE, and AA–AD defined in Figure 1) was obtained by scraping the silica gel plate based on a retention factor (Rf). The PLC results were visualized with a UV lamp (240–400 nm), and each area spot was categorized as one fraction.

### 2.3. Primary No-Choice Feeding Tests against R. speratus

The crude extractives dissolved in each solvent at a 2% (w/v) concentration were used in no-choice feeding tests. An aliquot (30 μL) of each sample was placed onto a paper disc (30 mg, 8 mm in diameter, thick type; ADVANTEC TOYO, Tokyo, Japan), which was dried at 60 °C for 24 h to remove the solvent. No-choice feeding tests were performed using 50 workers and five soldiers of *R. speratus* in an incubator at 28 °C for 14 days. After this time, paper discs were taken out, cleaned from debris, oven-dried at 60 °C for 24 h, and finally weighed to calculate mass loss [9]. As a control, untreated paper discs (PDUs) were dried in the same manner as the discs with the extractives. To measure termiticidal activity, dead termites were counted. This test was carried out using *R. speratus* maintained at the University of Tsukuba. Three replicates were performed for each sample.

### 2.4. No-Choice Feeding Tests against R. speratus and C. formosanus Using Fractions of Teak Extractives

A paper disc was impregnated with 30 μL of each sample containing ethanol, chloroform, or acetone extractives with an equal amount of MAQ (22 μg per paper disc). In addition, a paper disc was impregnated with a fraction for the 14 fractions from the three extractives. Disks were then used in no-choice feeding tests, performed as described in Section 2.3., with 50 workers and five soldiers of *R. speratus* or *C. formosanus* individuals originating from the colonies maintained at the University of Tsukuba and FFPRI, respectively.

### 2.5. Statistical Analysis

The effects of MAQ on mass loss and termite mortality were analyzed with an analysis of variance (ANOVA), and samples showing significant differences (*p* < 0.05) were analyzed using Duncan’s post hoc test (α = 0.05). All analyses were performed using IBM SPSS version 22 (IBM Japan, Tokyo, Japan).

### 2.6. Gas Chromatography (GC) and Pyrolysis-Gas Chromatography–Mass Spectroscopy (Py-GC/MS)

The amounts of MAQ in the extractives (including extractives’ fractions) were analyzed using a GC-17A gas chromatograph (Shimadzu, Japan), equipped with a DB-1 column (30 m × 0.25 mm i.d., film thickness, 0.25 μm) and a flame ionization detector using helium as the carrier gas. The injection temperature was 300 °C and the split ratio was 50:1. The temperature profile for GC analysis was as follows: 5 min at 160 °C, 12 min at 160–280 °C (10 °C/min), and 4 min at 280 °C. The MAQ content of the extractives was determined by comparison with a standard prepared from the authentic compound.

The contents of MAQ in raw materials and extracted residues were analyzed by pyrolysis-gas chromatography-mass spectrometry (PyGC/MS). After *n*-eicosane as an internal standard was added, wood powders or extracted residues (150–200 μg, measured with a micro balance) were wrapped with a pyrofoil and pyrolyzed at 500 °C for 4 s using a JHP-5 pyrolyzer (Japan Analytical Industry, Tokyo, Japan), which was interfaced (interface temperature 250 °C) with a GC/MS system QP-5050 (Shimadzu, Kyoto, Japan) equipped with an HP-1MS column (30 m × 0.25 mm i.d. film thickness, 1.0 μm), with electron impact of 70 eV and helium as a carrier gas. The temperature profile for GC was as follows: 1 min at 50 °C, 5 min at 50–280 °C (5 °C/min), and 13 min at 280 °C. Products resulting from the pyrolysis were identified by comparing their retention times and mass spectra data with those obtained for authentic compounds and published data [10].

## 3. Results

### 3.1. Extraction, Fractionation, and Determination of MAQ Content

Extractives contents in teak wood are related to tree age, which is one of the most important factors affecting the natural durability of wood [11]. In teak wood, the number of black stripes increases with increasing age and represents a type of defense mechanism that protects teak wood from insects and fungi [2]. In addition to tree age, longitudinal variation, geographical location, environmental conditions, and silvicultural activities can influence heartwood extractives, color properties, and durability [2]. The MAQ content determined in the present study (Table 1) was similar to previous reports indicating that teak wood extractives contain 0.3%–0.9% of MAQ [5,12,13].

The means of crude extractives and MAQ yields of the wood powders of *T. grandis* individually Soxhlet-extracted with ethanol, chloroform, and acetone are shown in Table 1. MAQ yields in the extractives were determined using GC, whereas the extracted residues were analyzed by Py-GC/MS. Chloroform resulted in the highest extractive yield among the three solvents. In the PLC of the crude extractives, Rf values of the main fractions that included MAQ were 0.52–0.56, which were similar to the Rf value of the authentic MAQ compound (Table 2).

**Figure 2:**
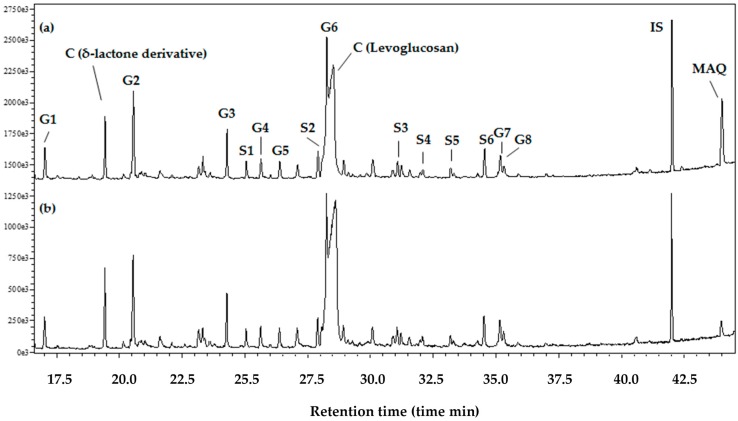
Pyrogram obtained in the Py-GC/MS of *T. grandis* heartwood (**a**) and chloroform-extracted residues (**b**). Legends: IS = internal standard (*n*-eicosane); G1 = guaiacol; G2 = 4-methylguaiacol; G3 = 4-vinylguaiacol; G4 = eugenol; G5 = vanillin; G6 = *trans*-isoeugenol; G7 = coniferaldehyde; G8 = trans-coniferyl alcohol; S1 = syringol; S2 = 4-methylsyringol; S3 = 4-vinylsyringol; S4 = 4-allylsyringol; S5 = syringaldehyde; S6 = *trans*-4-propenylsyringol; G1–G8 = pyrolysis products from guaiacyl lignin unit; S1–S6 = pyrolysis products from syringyl lignin unit; C = pyrolysis products from carbohydrate; and MAQ = 2-methylanthraquinone.

The Py-GC/MS generated a MAQ peak and pyrolysis products (G1–G8, S1–S6, C) peaks corresponding to lignin and carbohydrates (Figure 2). Chloroform-extraction of the raw material clearly decreased the MAQ peak, while the lignin and carbohydrate pyrolysis product peaks were retained.

### 3.2. Primary No-Choice Feeding Test of Crude Extractives in R. speratus

**Figure 3:**
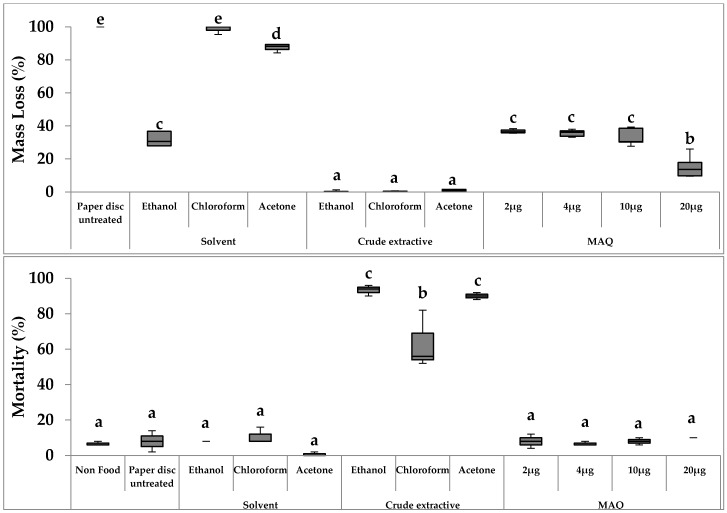
Box plot of mass loss and mortality of *R. speratus* in no-choice feeding tests using filter paper discs treated with *T. grandis* crude extractives and MAQ. Notes: Treatments with the same letters in the same graphic are not statistically different at *p* < 0.05, as determined by Duncan’s test; Dosages of the extractives and MAQ are indicated in Table 1.

The results of the primary no-choice feeding tests are shown in Figure 3. The mass loss of paper disks and termite mortality were determined after 14 days of observations. Mass loss in ethanol, chloroform, and acetone extractives was less than 1%, whereas MAQ dosage (10, 6.8, and 15 μg) differed among the three crude extractives (Table 1). In addition, mass loss using 2–10 μg MAQ was approximately 40% whereas that using 20 μg MAQ was 20%. Ethanol and acetone crude extractives presented the highest levels of termite mortality (93% and 90%, respectively), whereas chloroform crude extractives produced a moderate level of mortality (63%). The treatment using 20 μg of MAQ resulted in a lower termite mortality (approximately 10%) than that with the chloroform extractives containing using 6.8 μg MAQ. Crude extractives had marked effects on no-choice feeding tests but mass loss and mortality were not directly related to the MAQ dosage, in the 2–20 μg range.

ANOVA indicated significant differences (*p* = 0.001) in the mean of mass loss between test and control treatments.

### 3.3. No-Choice Feeding Tests of Crude Extractives in R. speratus and C. formosanus

#### 3.3.1. Mass Loss

To further clarify the effects of MAQ, no-choice feeding tests were conducted using *R. speratus* and *C. formosanus*. The concentrations of crude extractives were adjusted to obtain an equal amount of MAQ (22 μg per paper disc). In addition, PLC fractions from crude extractives (Table 2) were used in no-choice feeding tests. This experiment evaluated the effect of other compounds in the crude extractives on mass loss and termite mortality.

**Figure 4:**
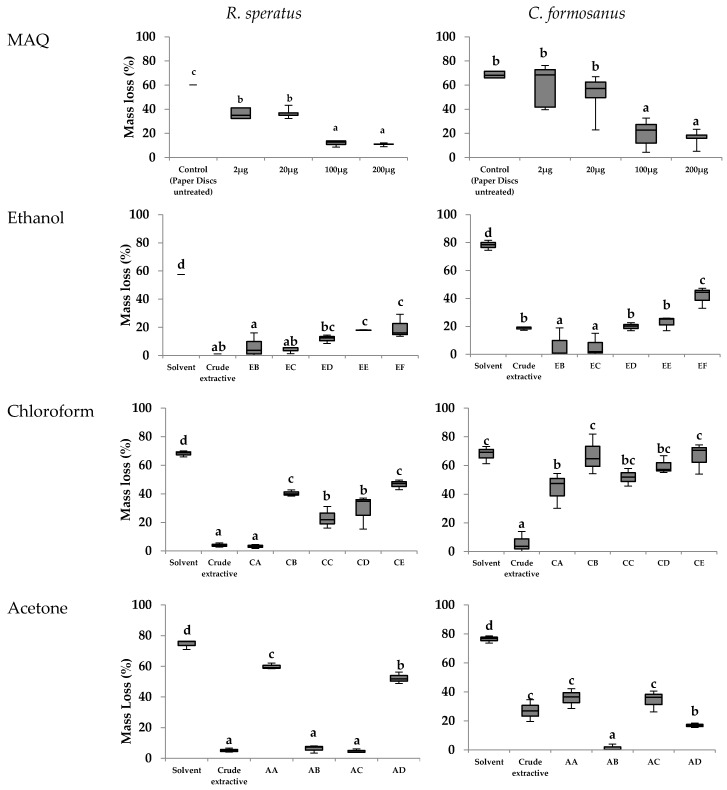
Box plot of the mass loss registered in *R. speratus* and *C. formosanus* no-choice feeding tests using fractions of *T. grandis* crude extractives and MAQ. Notes: Treatments with the same letters in the same graphic are not statistically different at *p* < 0.05, as determined by Duncan’s test; EB-EF, CA-CE, and AA-AD are referred to Figure 1 and Table 2; Dosages of extractives and MAQ are indicated in Table 2.

Figure 4 shows the mass loss for *R. speratus* and *C. formosanus* after 14 days of observations. A close examination of crude extractives containing 22 μg MAQ showed similar mass loss using ethanol, chloroform, and acetone extractives in *R. speratus* tests (2.4%, 4.1%, and 5.2%, respectively). Mass losses with *C. formosanus* tests tended to be higher than those with *R. speratus* (18.7%, 5.9%, and 27.1% in ethanol, chloroform, and acetone extractives, respectively).

Low mass losses were obtained for the fractions, EB (55 μg MAQ), EC (16 μg MAQ), CA (0.8 μg MAQ), AB (162 μg MAQ), and AC (7.6 μg MAQ) in *R. speratus* tests. In contrast, mass losses of these fractions in *C. formosanus* tests tended to be high: notable mass loss values were obtained for EB (6.7%), EC (5.7%), and AB (1.4%).

For comparison, paper discs were also impregnated with a standard MAQ compound (2, 20, 100, and 200 μg per paper disc). Significant differences were observed in both subterranean termites using 100 and 200 μg MAQ compound impregnations, with mass losses in the 10%–20% range.

The ANOVA revealed significant differences in mass loss among the *R. speratus* tests using MAQ, and ethanol and chloroform extractives (*p*: 0.078, 0.002, and 0.005, respectively), but not using acetone extractives (*p*: 0.323). Significant differences in mass loss were found with *C. formosanus* tests for MAQ and ethanol extractives (*p*: 0.014 and 0.021), but not for chloroform or acetone extractives (*p*: 0.477 and 0.224).

#### 3.3.2. Termite Mortality

**Figure 5:**
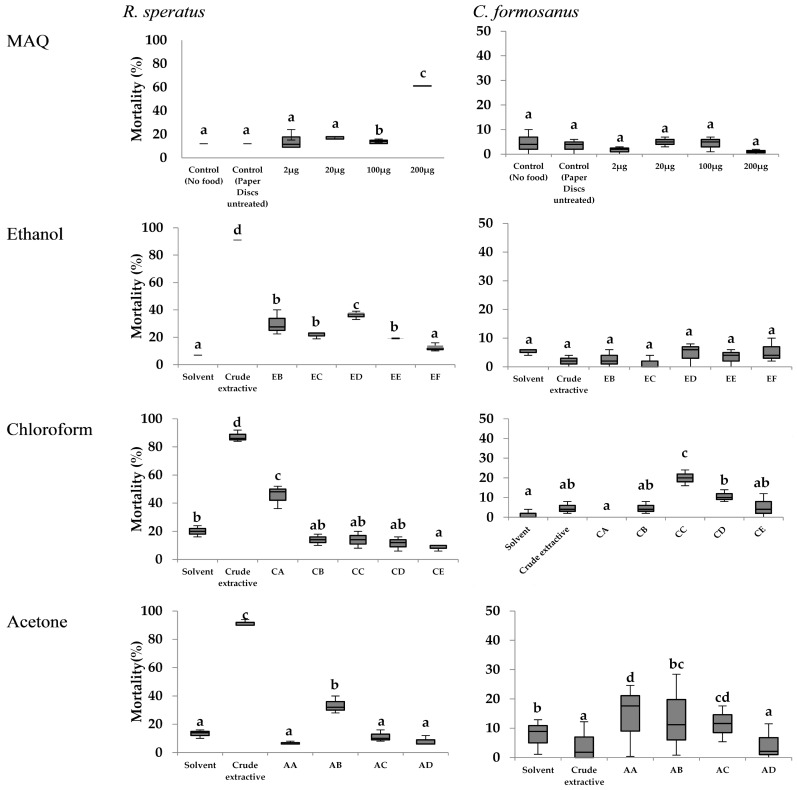
Box plot of the mortality observed for *R. speratus* and *C. formosanus* in no-choice feeding tests using fractions of *T. grandis* crude extractives and MAQ. Notes: Treatments with the same letters in the same graphic are not statistically different at *p* < 0.05, as determined by Duncan’s test; EB-EF, CA-CE, and AA-AD are referred to Figure 1 and Table 2; Dosages of extractives and MAQ are indicated in Table 2.

Mortality of both *R. speratus* and *C. formosanus* in the no-choice feeding test are summarized in Figure 5. There were significant mortalities in tests of the extractives with 22 μg MAQ for *R. speratus* using the ethanol, chloroform, and acetone extractives (92.0%, 87.3%, and 91.3%, respectively). In contrast, the extractives had no effect on the mortality against *C. formosanus* (less than 5%).

Notably high mortalities were caused by exposure to CA (0.8 μg MAQ) or AB (162 μg MAQ) fractions on *R. speratus* and by CC (none MAQ) on *C. formosanus*. These results did not appear to be related to MAQ dosages. Meanwhile, the MAQ control test indicated that 200 μg was the only MAQ dosage that significantly affected *R. speratus* mortality, whereas none of the dosages affected *C. formosanus* mortality.

There were no significant differences in termite mortality among tests (MAQ, ethanol, chloroform, and acetone extractives) for both termites (*p* values for *R. speratus*: 0.413, 0.574, 0.479, 0.182, respectively; *p* values for *C. formosanus*: 0.241, 0.327, 0.160, and 0.674, respectively).

## 4. Discussion

### 4.1. Determination of MAQ in Teak Wood and PLC Fractions

The MAQ peak in the Py-GC/MS analysis was detected in addition to pyrolysis products from lignin and carbohydrates [14]. The MAQ peak detected at the end of the chromatogram was successfully determined in this study using a calibration line, indicating a 0.21% yield based on wood weight (Figure 2). Carbohydrates are readily fragmented into smaller compounds, making it difficult to determine their origin (cellulose and hemicelluloses), but levoglucosan was detected in the middle of the chromatogram, along with lignin pyrolysis products. This is consistent with the range of MAQ content (0.2%–1.1%) determined in a previous study using a Py-GC/flame ionization detector [15].

The MAQ yield of the remaining residues after wood powder extraction, as determined by Py-GC/MS, ranged from 0.04% to 0.05%. The MAQ yield of ethanol extractives was 0.11%, and the sum of the yields was 0.16%, meaning there was a difference between the content of raw material (0.21%) and the calculated yield (0.16%). This difference might be owing to the Py-GC/MS not requiring sample preparation and being more accurate than GC. As MAQ and other anthraquinone derivatives might play a role in teak wood durability, the use of Py-GC/MS is useful for detecting compounds that might have toxicity and feed-deterrent effects.

### 4.2. Primary No-Choice Feeding Tests of Crude Extractives against R. speratus

The primary no-choice feeding tests using *R. speratus* revealed that crude extractives of *T. grandis* clearly displayed a feeding deterrent effect, as indicated by the highly significant difference in the mass loss of paper discs (Figure 3). Meanwhile, control tests using a standard MAQ compound indicated that MAQ (2–20 μg) dosage was not directly related to termite feeding activity. Moreover, the higher termite mortalities found in crude extractives tests than in control solvents (blank tests) and MAQ compound tests demonstrated their effect. Termite mortalities caused by ethanol and acetone extractives were high, whereas those caused by chloroform were moderate.

Lukmandaru and Ogiyama [3] suggested that the high termite mortality observed in teak wood extractives might be due to MAQ, which was proposed to have both antifeedant activity and toxicity. Thus, the effect of MAQ dosage on termite resistance should be further investigated. Differences in toxicity might also be related to termite species [16]. We used two species of subterranean termites, *R. speratus* and *C. formosanus*, which are known to cause extensive damage to wood construction [16]. In addition, as *C. formosanus* is a native pest, it might provide valuable information regarding the natural durability of teak wood.

### 4.3. Toxicity and the Feeding Deterrent Effect of Wood Extractives (Including MAQ) against R. speratus and C. formosanus

#### 4.3.1. Effect of Crude Extractives

No-choice feeding tests of *T. grandis* crude extractives, including MAQ, clearly displayed feeding deterrent effects and toxicity against *R. speratus* and had a feeding deterrent effect against *C. formosanus* (Table 3). No-choice feeding tests using MAQ against *R. speratus* confirmed its feeding deterrent effects at 100–200 μg dosages and toxicity at 200 μg. For *C. formosanus*, the tests using MAQ also confirmed feeding deterrent effects at 100–200 μg, but not toxicity. These results suggest the amount of MAQ is not directly related to its feeding deterrent effect or toxicity and that *C. formosanus* is more resistant than *R. speratus*. These results concur with those from previous studies demonstrating that different termite species have different susceptibility and resistance towards wood extractives [16,17,18].

#### 4.3.2. Effect of the Fractions Derived from Crude Extractives

All ethanol extractive fractions (EB-EF) and two of the acetone extractive fractions (AB, AC) clearly displayed feeding deterrent effects against *R. speratus*, similar to that obtained in a crude extractives bioassay. EB-EE fractions and AB and AD fractions displayed feeding deterrent effects against *C. formosanus* similarly to that of crude extractives. The EC fraction, in particular, showed a higher effect than the crude extractive.

Previous studies [1,2] suggested that MAQ only affected the palatability of termites with regard to wood or merely discouraged termite feeding, and our findings are consistent with this suggestion. Although the effects of extractive compounds on termite resistance possibly vary with concentration, the no-choice feeding tests conducted in the present study clearly indicated the opposite. Toxicity and feed deterrent effects did not depend on MAQ dosages and were possibly derived from the coexistence of MAQ and other compounds in teak wood extractives, as MAQ alone did not cause toxicity or feed deterrent effects, except at very high dosages. Thus, the concentrations of other active compounds and the interaction between MAQ and other components in teak wood extractives might lead to the high feeding deterrent effect observed.

## 5. Conclusions

Ethanol, chloroform, and acetone extractives of *T. grandis* heartwood deterred feeding in *R. speratus* and *C. formosanus*. No-choice feeding tests clearly indicated that the toxicity and feed deterrent effects were independent of MAQ dosages, but were derived from the combination of teak wood extractives and small amounts of MAQ. The amount of MAQ in the extractives was not related to toxicity and the feed deterrent effect. *C. formosanus* was more tolerant to the extractives than *R. speratus*, and MAQ was not effective against this species. Interactions between MAQ and other components in teak wood extractives led to a high feeding deterrent effect.

## Figures and Tables

**Table 1:** Content of 2-methylanthraquinone (MAQ) in crude extractives and residues of *T. grandis* heartwood and the dosages of crude extractives and MAQ used in no-choice feeding tests.

Extraction Solvent	Crude Extractives Recovered (%) ^1^	MAQ Content in Crude Extractives (%) ^1^	MAQ Content in Extracted Residue (%) ^2^	Dosage in No-Choice Feeding ^3^	
				Crude Extractives (μg)	MAQ (μg)
Ethanol	6.5 ± 0.6	0.11 ± 0.01	0.05 ± 0.01	602	10
Chloroform	8.7 ± 0.2	0.10 ± 0.01	0.05 ± 0.02	606	6.8
Acetone	6.6 ± 0.2	0.15 ± 0.01	0.04 ± 0.01	666	15

^1^ Mean ± standard deviation analyzed by GC, based on wood; ^2^ Mean ± standard deviation analyzed by Py-GC/MS, based on residue; ^3^ No-choice feeding test against *R. speratus*.

**Table 2:** Fractions obtained from crude extractives by preparative layer chromatography (PLC).

Fraction ^1^	Rf	Extractives Weight (μg)	MAQ Weight (μg)	Dosage in No-Choice Feeding Test ^2^	
				Extractives (μg)	MAQ (μg)
**Ethanol crude extractives**				1296	22
EB	0.52 ± 0.16	5590	468	656	55
EC	0.27 ± 0.01	2470	64.4	600	16
ED	0.21 ± 0.05	2890	2.55	573	0.5
EE	0.07 ± 0.04	10,300	2.37	687	0.2
EF	0.00 ± 0.01	30,400	2.37	779	0.1
**Chloroform crude extractives**				1960	22
CA	0.55 ± 0.02	21,900	25.3	683	0.8
CB	0.41 ± 0.04	14,000	0.81	698	0
CC	0.34 ± 0.03	14,600	0.12	795	0
CD	0.06 ± 0.03	20,700	0.35	848	0
CE	0.00 ± 0.01	15,500	0.46	764	0
**Acetone crude extractives**				946	22
AA	0.69 ± 0.06	2540	3.06	585	0.7
AB	0.56 ± 0.04	3730	887	681	162
AC	0.24 ± 0.16	7450	68.6	828	7.6
AD	0.00 ± 0.01	10,120	26.5	707	1.9

Legends: Rf = retention factor; ^1^ Amounts of ethanol, chloroform, and acetone crude extractives using PLC were 108, 103, and 111 mg, respectively; ^2^ No-choice feeding test against *R. speratus* and *C. formosanus*.

**Table 3:** Effects of MAQ and *T. grandis* wood extractives on toxicity and the feeding deterrence against the subterranean termites *R. speratus* and *C. formosanus*.

Fraction	Dosage		*R. speratus*		*C. formosanus*	
	Extractives (μg)	MAQ (μg)	Toxicity	Feeding Deterrent	Toxicity	Feeding Deterrent
MAQ	0	200	++	++	-	++
MAQ	0	100	-	++	-	++
MAQ	0	20	-	+	-	-
Ethanol crude extractives	1296	22	++	++	-	++
EB	656	55	+	++	-	++
EC	600	16	+	++	-	++
ED	573	0.5	++	++	-	++
EE	687	0.2	-	++	-	++
EF	779	0.1	-	+	-	+
Chloroform crude extractives	1960	22	++	++	-	++
CA	683	0.8	++	++	-	+
CB	698	0	-	-	-	+
CC	795	0	-	+	-	-
CD	848	0	-	+	-	-
CE	764	0	-	-	-	-
Acetone crude extractives	946	22	++	++	-	+
AA	585	0.7	-	-	-	+
AB	681	162	+	++	-	++
AC	828	7.6	-	++	-	+
AD	707	1.9	-	-	-	++

Legend: Mortality based on control (no food); ++: more than two times; +: 1–2 times; Mass loss based on solvent; ++: less than 30%; +: 30%–60%.
